# Integrating trajectory inference and self-explainable predictive models to explore cell state transitions in breast cancer at single-cell resolution

**DOI:** 10.3389/fbinf.2026.1672671

**Published:** 2026-03-04

**Authors:** Vanessa Verrina, Marianna Talia, Eugenio Cesario, Santina Capalbo, Domenica Scordamaglia, Rosamaria Lappano, Anna Maria Miglietta, Marcello Maggiolini, Sabrina Giordano

**Affiliations:** 1 Department of Economics, Statistics and Finance “Giovanni Anania”, University of Calabria, Rende, Italy; 2 Department of Pharmacy, Health and Nutritional Sciences, University of Calabria, Rende, Italy; 3 Department of Cultures, Education and Society, University of Calabria, Rende, Italy; 4 Department of Computer Science, Modeling, Electronics and Systems Engineering, University of Calabria, Rende, Italy; 5 Department of Medicine and Surgery, “Kore” University of Enna, Enna, Italy; 6 Department of Experimental and Clinical Medicine, University “Magna Græcia” of Catanzaro, Catanzaro, Italy; 7 Breast and General Surgery Unit, Annunziata Hospital Cosenza, Cosenza, Italy

**Keywords:** breast cancer, interpretability, single-cell RNA sequencing, statistical learning, trajectory inference

## Abstract

**Introduction:**

Breast cancer is characterized by a highly heterogeneous cellular environment composed of diverse malignant clones and components of the tumor microenvironment (TME) that collectively influence disease progression. Single-cell RNA sequencing (scRNA-seq) offers a powerful tool to dissect this complexity, enabling high-resolution characterization of tumor heterogeneity and functional interactions within the TME. Moreover, it supports the discovery of clinically relevant subpopulations and potential therapeutic targets.

**Methods:**

In this study, we present a novel scRNA-seq dataset from an infiltrating ductal breast cancer, profiling over 5,000 cells and identifying six distinct clusters spanning cancer and TME populations. To explore the molecular drivers of cell state transitions, we integrate pseudotime trajectory inference with interpretable, tree-based machine learning. This combined approach enables the identification of key genes and expression thresholds associated with dynamic phenotypic shifts.

**Results:**

Our analysis identified six distinct cellular clusters representing both malignant and TME populations. The integration of pseudotime inference with interpretable machine learning uncovered key genes and specific expression thresholds associated with transcriptional reprogramming and dynamic phenotypic transitions during tumor evolution.

**Discussion:**

Unlike black-box models, our framework provides transparent, rule-based insights into transcriptional reprogramming processes underlying tumor progression. The resulting dataset, together with an accessible and transparent analytical pipeline, represents a valuable resource for the breast cancer research community and establishes a foundation for future studies aimed at refining molecular classification and advancing precision therapy development.

## 1 Introduction

Cancer has been defined as an evolutionary disease that arises from a redundant iteration of clonal expansion, genomic alterations and clonal selection (Black and McGranahan, 2021; Greaves and Maley, 2012). The genomic instability that characterizes cancer cells drives intratumoral heterogeneity at both spatial and temporal levels, therefore generating subpopulations of cells with distinct omics profiles and biological responses to treatment (Dagogo-Jack et al., 2018; Chen et al., 2024). This high plasticity can also lead to intertumoral heterogeneity, which is described by the alterations observed among tumors affecting the same tissues of diverse patients (Prasetyanti and Medema, 2017). Adding a further layer of complexity, the tumor microenvironment (TME) can be defined as the dynamic and heterogeneous milieu containing cancer cells surrounded by immune, stromal, and endothelial cells, as well as extracellular matrix (ECM) components and soluble mediators including cytokines, chemokines, and growth factors. This multifaceted ecosystem continuously evolves during tumor progression and exerts a profound influence on cancer cell growth, survival, invasion, and metastatic dissemination (Anderson and Simon, 2020; Zhao et al., 2023; Lappano et al., 2020).

Despite advances in earlier detection and more effective treatment, breast cancer remains the most common malignancy that affects women worldwide and continues to represent a substantial global health concern (Giaquinto et al., 2024). Breast cancer cells are characterized by high heterogeneity on the basis of their multi-omic profile and proliferative, invasive, self-renewal and differentiation potential (Guo et al., 2023; Lüönd et al., 2021). Transcriptomic studies have helped to partially unravel this complexity by identifying intrinsic molecular subtypes of breast cancer, each exhibiting unique biological behaviors, clinical trajectories and therapeutic susceptibilities (Nolan et al., 2023; Perou et al., 2000; Sørlie et al., 2001). Molecular-level evaluation of the TME in breast cancer has introduced a further entanglement, particularly in elucidating its influence on therapeutic efficacy and patient outcomes (Sammut et al., 2022). The TME encompasses a diverse array of components and their dynamic interactions with cancer cells, all of which play a critical role in shaping disease progression and treatment response (Ding et al., 2020; Jovic et al., 2022). Understanding these interactions at high resolution enables deeper insights into key biological processes, including tumor evolution, immune cell infiltration, and mechanisms of drug resistance (Tang et al., 2009; Tang et al., 2010; Trapnell, 2015).

In this context, deciphering breast cancer heterogeneity at single-cell resolution has emerged as a critical objective in the era of precision medicine, offering unprecedented resolution to interrogate the intricate cellular and molecular architecture of individual tumors, thus enabling the formulation of patient-specific therapeutic regimens based on the nuanced biology of their disease (Pal et al., 2021; Wu et al., 2021a). Single-cell RNA sequencing (scRNA-seq) enables the identification of rare but clinically consequential subpopulations—such as cancer stem-like cells and drug-resistant clones—that may drive tumor persistence, metastatic dissemination, and therapeutic failure (Grosselin et al., 2019). scRNA-seq has also revealed new insights into the epithelial to mesenchymal transition (EMT) in cancer, challenging the notion of a simple switch from epithelial to mesenchymal states, and instead revealing that it is a dynamic process with diverse hybrid cellular intermediates. During EMT, epithelial cells lose stable junctions and acquire migratory and invasive features through the activation of diverse gene transcriptional programs (Cook and Vanderhyden, 2020; Malagoli Tagliazucchi et al., 2023; Murayama and Matsuda, 2022).

Strategic targeting of these phenotypically and functionally distinct cellular compartments holds significant promise for the development of next-generation, mechanism-informed cancer therapeutics.

## 2 Results

**FIGURE 1:**
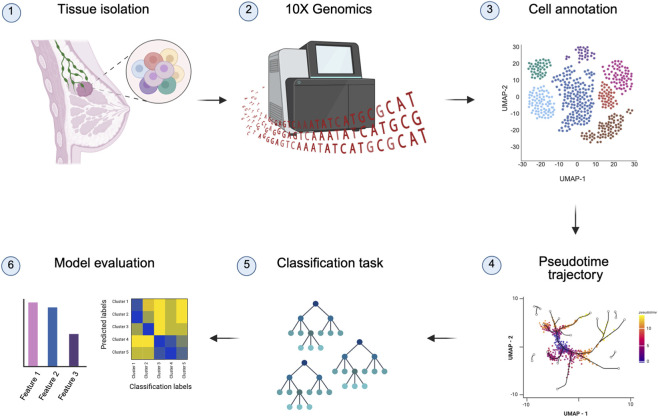
Overview of the single-cell RNA-seq data analysis workflow. The end-to-end process includes the following steps: (1) harvesting breast cancer samples, (2) constructing and sequencing scRNA-seq libraries using 10X Genomics, (3) computational data processing (including clustering and cell annotation), (4) pseudotime trajectory analysis, (5) implementing machine learning-based classification models, and (6) evaluating model interpretability for gene selection. Created with BioRender.com.

An overview of the scRNA-seq analysis of breast cancer tissue is provided in Figure 1. The workflow begins with the collection of a primary tumor tissue sample from a breast cancer patient. This is followed by the construction of a scRNA-seq library using the 10x Genomics Chromium platform, which enables high-throughput single-cell transcriptomic profiling. After sequencing, the raw data undergo standard preprocessing steps, including quality control (QC), normalization, and dimensionality reduction. Subsequently, cells are annotated into distinct cell types or states based on known marker genes and clustering techniques. Trajectory inference is then performed to capture dynamic cellular processes such as differentiation or tumor progression. The processed data is subsequently used to train machine learning models aimed at classifying specific cell populations or identifying key marker genes associated with disease states. Finally, the performance of these models is evaluated, and their transparent, rule-based structure is exploited to interpret key features and decision boundaries, providing biologically meaningful insights into the underlying cellular heterogeneity of breast cancer.

### 2.1 Data processing and cell type characterization from scRNA-seq

**FIGURE 2:**
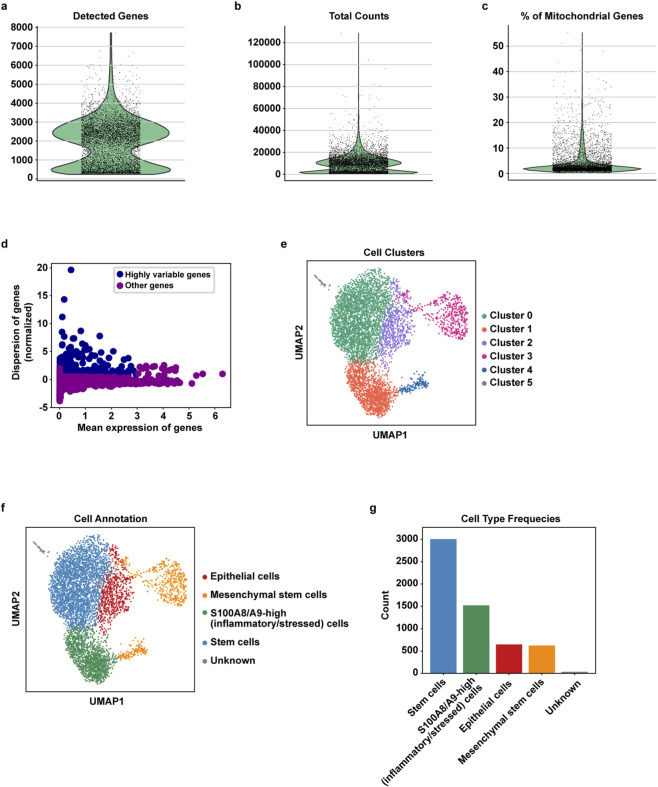
Preprocessing of scRNA-seq data. **(a)** Violin plot depicting the number of detected genes for each cell. **(b)** Violin plot of the total gene counts. **(c)** Violin plot showing the percentage of mitochondrial genes in each cell shows that most cells contain less than 5% mitochondrial genes. **(d)** Scatter plot of highly variable genes (blue dots) compared to other genes (magenta dots). **(e)** UMAP representation illustrates the six clusters identified using the Leiden algorithm at a resolution of 0.3. **(f)** UMAP visualization of cell annotation results using the SCSA tool. The analysis identified four distinct populations, with groups 3 and 4 both classified as mesenchymal cells. **(g)** Bar chart showing the size of each cell group.

The ductal breast carcinoma scRNA-seq data were first processed for QC. In Figure 2, key metrics such as the number of unique genes detected (Figure 2a), total gene counts (Figure 2b), and the proportion of mitochondrial gene expression per cell (Figure 2c) are shown. Based on these QC metrics, cell filtering was performed as described in Section 4. Furthermore, features exhibiting high cell-to-cell variability were identified and filtered to ensure data quality (Figure 2d). After QC, 5,834 high-quality cells were retained for downstream analyses.

To explore the cellular composition of the tumor sample, we performed unsupervised clustering on the high-quality single-cell transcriptomes. This analysis identified six transcriptionally distinct clusters (Figure 2e). The size of each cluster ranged from 34 to 3,006 cells, reflecting the heterogeneity of the tumor ecosystem.

Each cluster was annotated based on the expression of canonical marker genes, allowing the identification of major cell types within both the tumor and its microenvironment. Cell type annotation was performed using SCSA, applying thresholds based on LFC ⩾ 1 and P < 0.05, which led to the identification of five distinct cell populations (Figure 2f). Specifically, cluster 0 was identified as stem cells, cluster 2 as epithelial cells, clusters 3 and 4 as mesenchymal stem cells. Cluster 1 was manually labeled as “S100A8/A9-high (inflammatory/stressed) cells” based on high transcriptional levels of S100A8 and S100A9 as well as the acute phase markers SAA1 and SAA2, reflecting a putative inflammatory and stress-responsive cellular phenotype (Malagoli Tagliazucchi et al., 2023; Murayama and Matsuda, 2022; Wu and Dai, 2017). In addition, cells belonging to cluster 5 were unable to be clearly annotated due to an ambiguous expression profile, thus remaining classified as “Unknown” (Figure 2g).

Notably, some of the identified populations represent key cellular components of the TME, which is actively shaped by cancer cells and, in turn, exerts a profound influence on tumor progression (Ugel et al., 2021). Mesenchymal stem cells (clusters 3 and 4) are known to provide structural support to the tumor and to secrete paracrine factors that may influence both neoplastic cells and TME components. The presence of stem-like populations (cluster 0) reflects the plasticity of the TME, potentially sustaining tumor heterogeneity and therapy resistance (Prasetyanti and Medema, 2017). Collectively, these findings capture the complexity of the interactions that may occur between tumor cells and the surrounding stroma. A summary of the clusters and their corresponding cell type annotations is presented in Table 1.

**TABLE 1:** Cluster annotations based on canonical marker gene expression. Cluster sizes refer to the number of cells assigned after quality control (QC) and unsupervised clustering.

Cluster ID	Number of cells	Putative cell type	Marker genes
0	3,006	Stem cells	MYC, CD44, KRT15, EPCAM, ITGA6, TP63
1	1,522	S100A8/A9-high (inflammatory/stressed) cells	S100A8, S100A9, SAA1, SAA2
2	649	Epithelial cells	CDH1, KRT7, CLDN1, OCLN, MUC1, KRT8, KRT17, KRT5, KRT16, KRT6C, KRT6A, KRT6B
3	484	Mesenchymal stem cells	VIM, S100A4, FN1, COL1A1, COL1A2
4	139	Mesenchymal stem cells	VIM, S100A4, FN1, COL1A1, COL1A2
5	34	Unknown	-

### 2.2 Identification of differentially expressed genes (DEGs)

**FIGURE 3:**
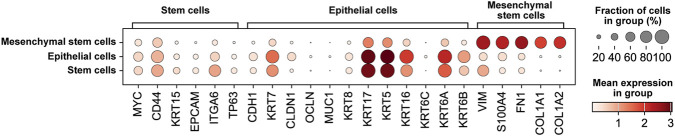
Cell type annotation based on canonical and dataset-specific marker genes. Dot plot summarizing the average expression of key marker genes across all identified clusters. The size of each dot reflects the proportion of cells in the cluster expressing the marker, while the color intensity indicates the average expression level. This visualization highlights the specificity and distribution of marker expression across clusters and provides the basis for assigning cell type identities within the tumor microenvironment.

We then performed an in-depth comparison and analysis of the five remaining populations. We explored the expression of prototypical markers within the diverse cellular types, as shown in Figure 3.

Stem cells exhibit high expression of EPCAM, CD44, ITGA6, and TP63 (Figure 3), which are well-established stemness markers in breast cancer. These genes are widely recognized for their role in maintaining self-renewal, differentiation capacity, and resistance to conventional therapies (Zhao et al., 2017; Li et al., 2023). In particular, the expression of EPCAM (Epithelial Cell Adhesion Molecule) and the transmembrane glycoprotein CD44 is associated with enhanced tumorigenic and metastatic potential in breast cancer stem cells (Trzpis et al., 2007; Taurin and Alkhalifa, 2020). ITGA6 (Integrin Alpha 6) plays a crucial role in anchoring stem cells to the basement membrane and is associated with high clonogenic and tumor-initiating capacity in breast cancer, while the transcription factor TP63 is known for its critical role in maintaining cell stemness (Stingl et al., 2006; Galoczova et al., 2018). Epithelial cells are characterized by elevated levels of CDH1, CLDN1, MUC1, KRT7, and OCLN (Figure 3), which collectively contribute to the maintenance of epithelial integrity, polarity, and functional differentiation (Famta et al., 2025; Chen et al., 2021; Perelmuter et al., 2024; Nguyen et al., 2018). Specifically, the so-called cell adhesion gene CDH1 encodes the major cadherin in luminal epithelial cells, named E-cadherin, which is a hallmark of epithelial identity as it mediates cell-cell adhesion and suppresses epithelial-to-mesenchymal transition (EMT) (van Roy and Berx, 2008; Mendonsa et al., 2018; Zihni et al., 2016). CLDN1 (claudin-1) and OCLN (occludin) are essential transmembrane components of tight junctions, playing a pivotal role in barrier functions and maintenance of apical-basal polarity (Chen et al., 2021; Sandilands et al., 2013; Zhao et al., 2012). Similarly, MUC1 encodes a membrane-bound mucin (glycoprotein mucin 1) involved either in protecting epithelial surfaces by serving as a physical barrier in normal cells or in modulating intracellular signaling processes in cancer cells toward their acquisition of aggressive features (Wang et al., 2018; Timperi et al., 2022), while KRT7 is a type II intermediate filament cytokeratin (keratin 7) that contributes to cytoskeletal organization (Gu et al., 2024; Arai et al., 2008). Mesenchymal cells, in contrast, are characterized by the specific expression of genes involved in structural remodeling and EMT, including FN1 (fibronectin 1), S100A4, VIM (vimentin), COL1A1, COL1A2, and FAP (Fibroblast Activation Protein) (Figure 3) (Famta et al., 2025; Friedman et al., 2020; Cords et al., 2023). In particular, these genes play a central role in regulating mesenchymal identity, motility, and ECM dynamics toward the acquisition of an invasive cancer phenotype.

This distinct pattern of marker expression across cell populations not only validates our clustering analysis, but also underscores the cellular heterogeneity of the breast cancer microenvironment. Furthermore, the identification of population-specific molecular signatures provides a biological framework for interpreting pseudotime trajectories, enabling the detection of transitional states and potential lineage relationships toward a deeper understanding of tumor evolution and heterogeneity.

### 2.3 Reconstructing cellular differentiation trajectories

Single-cell RNA sequencing captures cells at diverse transcriptional states, which can be computationally ordered to estimate dynamic biological processes such as differentiation, activation, or fate transitions. Trajectory inference algorithms, including Monocle, Slingshot and PAGA (Trapnell et al., 2014; Street et al., 2018; Deconinck et al., 2021), reconstruct these processes by arranging cells along a pseudotime axis, under the assumption that transcriptomic heterogeneity reflects an underlying continuous progression of states. Applying trajectory inference to a single static dataset assumes that such dynamic processes are captured among the sampled cells. Within this framework, we used PAGA to infer pseudotemporal ordering and identify potential lineage relationships between cell populations.

**FIGURE 4:**
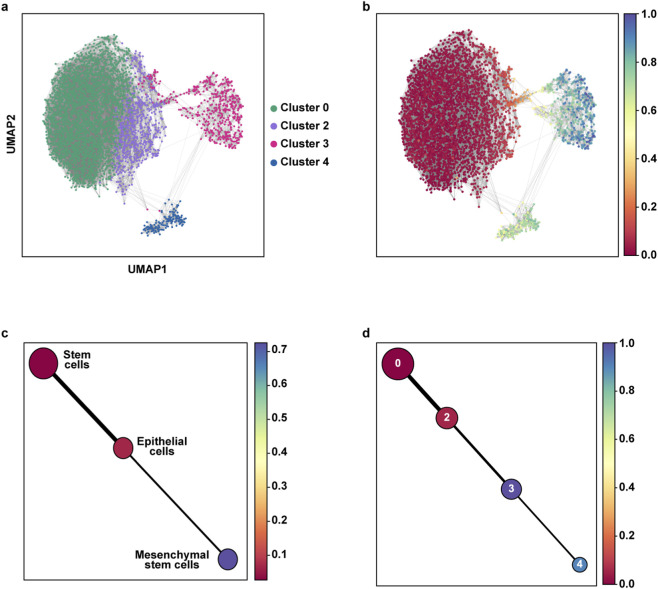
Diffusion pseudotime (DPT) analysis of cell populations. **(a)** UMAP representation of the selected cell populations, colored by cluster identity. **(b)** UMAP projection showing the DPT values for each cell; the color bar represents pseudotime, with earlier states in red and later states in blue. **(c)** Partition-based graph abstraction (PAGA) visualization of DPT highlighting major cell types. **(d)** PAGA representation highlighting individual clusters along the normalized inferred trajectory.

We excluded cluster 5 from downstream analysis due to its ambiguous identity, as the cells within this group could not be reliably classified based on known marker genes. Cluster 1, characterized by a pronounced S100A8/A9-high inflammatory/stress signature, was excluded from downstream analyses. Its annotation remains putative, and the transcriptional profile does not support a direct involvement in epithelial-to-mesenchymal transition processes typically associated with breast cancer progression. Pseudotemporal trajectory analysis was conducted on cells from clusters 0, 2, 3, and 4 to investigate the dynamic progression of cellular states during breast cancer evolution, as illustrated in Figure 4. The UMAP plot highlights the spatial distribution (Figure 4a) of the clusters and the calculated diffusion pseudotime for each cell (Figure 4b). On the other hand, the PAGA (Partition-based Graph Abstraction) graph outlines the connectivity and inferred trajectories between cell types, with nodes representing partitions, and edges weighted on the connectivity between them (Wolf et al., 2019) (Figures 4c,d). Stem cells were identified as the root node, characterized by the earliest pseudotime values and representing the starting point for epithelial cell differentiation. Pseudotime analysis revealed a well-defined transition from an epithelial to a mesenchymal phenotype (Figure 4c). Given the presence of two mesenchymal cell populations, we aimed at investigating the potential transition between these populations by performing the aforementioned analyses using clusters rather than cell types. As shown in Figure 4d, heterogeneity is observed between clusters 3 and 4, with mesenchymal cells of cluster 4 representing the terminally differentiated state. It should be noted that since pseudotime reflects a continuous measure applied to individual cells, whereas clusters are discrete groups, cells in cluster 3 can appear at later pseudotime values even though the global trajectory structure and marker patterns designate cluster 4 as the terminal branch.

Worthy, our findings are in line with previous studies showing that dissected EMT trajectories through pseudotime analyses of scRNA-seq data may enable the identification of succeeding cell states (Cook and Vanderhyden, 2020). In accordance with findings indicating that the number of possible partial EMT states may be likely innumerable (Cook and Vanderhyden, 2020), our results indicate that EMT is not a binary process, but a gradual and complex differentiation program, which is characterized by the transition of stem cells to epithelial intermediates ultimately progressing to diverse mesenchymal states.

### 2.4 From dynamics to decisions: linking cellular trajectories with gene-level insights

To identify the molecular drivers of cell state transitions, we employed classification tree models using annotated cell labels. This model reveals the hierarchical structure of decision rules that best separate all cell types based on gene expression. In contrast to black-box algorithms, classification trees are inherently interpretable, providing explicit decision rules that link gene expression thresholds to cell fate outcomes. This level of transparency is particularly valuable for biological interpretation and clinical translation, as it enables the identification of candidate regulatory genes and potential therapeutic targets. For these reasons, we adopted classification trees as the primary modelling framework in this study.

**FIGURE 5:**
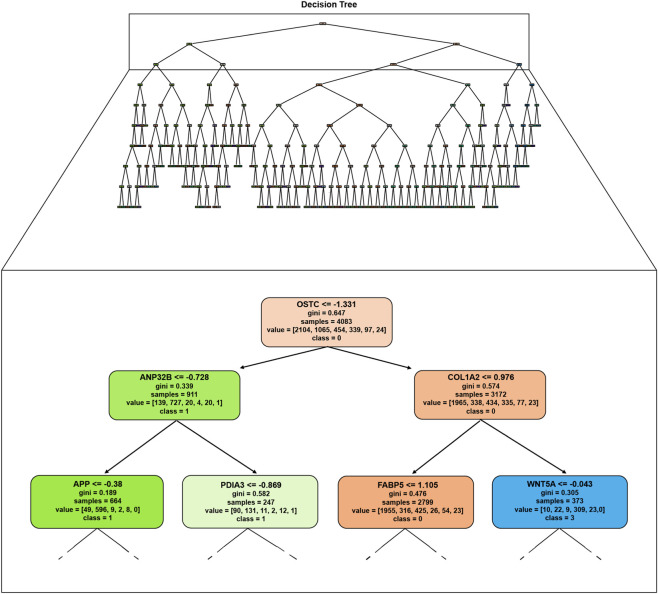
Classification tree trained on all the cell populations. To enhance readability, a zoomed-in view of the upper portion of the tree, including the root and the first two levels of child nodes, is provided. Each node represents the gene and its corresponding expression threshold that best separates the cells into distinct clusters, minimizing data impurity and improving classification accuracy.

Before the classification task, data preprocessing was carried out to ensure quality and relevance by filtering out noisy cells, low-quality or empty droplets, and non-informative gene features. The preprocessing procedure is detailed in Section 4.3. Thereafter, we trained a decision tree classifier with a maximum depth of 9 on all clusters, on the entire dataset. This initial step allowed us to obtain a global view of the major transcriptional features distinguishing the diverse cell populations within the tumor. Figure 5 shows the classification tree, along with a zoomed-in view of its upper portion, including the root node and the first two levels of child nodes. We can observe that each node shows several information, including the gene and its expression value that best separate or partition the given cells into individual cell types, aimed at reducing the impurity in the data. From the top part of this tree, only decision rules related to clusters 0, 1, and 3 are shown in Figure 5, while decision rules for slusters 2 and 4 occur in deeper nodes, which are not displayed for space constraints.

In this multi-class classification setting, we focused particularly on the first split of the decision tree, which represents the gene expression threshold providing the highest information gain across all classes. This top-level node defines the most critical bifurcation in the data, making it a particularly informative point of biological interpretation.

The decision rules derived from the model can be expressed as:

$$\text{Cluster}=\left\{\begin{array}{ll} 1, & \text{if }OSTC\leq -1.331\wedge ANP32B\leq -0.728\\ 0, & \text{if }OSTC>-1.331\wedge COL1A2\leq 0.976 \end{array}\right.$$

This structure highlights OSTC as the principal node splitting all cell types—suggesting it plays a central role in differentiating broad cellular states within the tumor. Additional branches involving ANP32B and COL1A2 provide finer resolution, reflecting downstream expression patterns relevant to more specific cell identities. While deeper nodes add granularity, the root split offers the most universal insight into transcriptomic divergence across the single-cell landscape.

**FIGURE 6:**
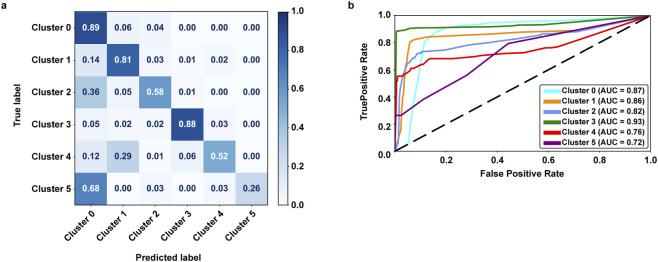
Classification performance of the decision tree model. **(a)** Confusion Matrix: Visual representation of the model’s classification performance across cell populations. Each entry C [i, j] indicates the percentage of cells from the true class i predicted as class j. **(b)** ROC Curves: Receiver Operating Characteristic curves for each cell population, illustrating the classifier’s discriminative power in distinguishing between cells.

To evaluate the performance of our decision tree classifier in distinguishing cellular states identified through pseudotime analysis, we examined both the confusion matrix and ROC curves (Figure 6).

The decision tree classifier demonstrated good predictive performance, achieving an average accuracy of 82% and AUC scores of 0.91 (micro-average value), underscoring its effectiveness in capturing key transcriptional features that discriminate between cellular states. This level of performance highlights the suitability of the model for single-cell transcriptomic applications, where interpretability and biological relevance are critical to understanding the complexity of tumor evolution.

Figure 6a reveals a high degree of classification accuracy for the majority of cell populations, particularly for Classes 0 and 3, where 89% and 88% of cells, respectively, were correctly assigned to their true labels. This suggests that these cellular states exhibit distinct and stable gene expression profiles, which are readily captured by the model’s decision rules.

However, classification performance decreased for clusters 4 and 5, which achieved correct classification rates of only 52% and 26%, respectively. The misclassification of cluster 5 cells—mostly predicted as cluster 0—may indicate a lack of defining transcriptional features or the insufficient representation in the dataset, possibly due to their rarity. Similarly, the confusion observed for cluster 4 highlights the challenge of resolving ambiguous or plastic phenotypes that may not exhibit discrete gene expression boundaries.

The ROC curve analysis (Figure 6b) further supports this interpretation. While clusters 0 through 3 yielded high AUC values (0.87, 0.86, 0.82 and 0.93, respectively), indicative of strong separability in the feature space, cluster 4 and 5 showed lower AUCs (0.76 and 0.72, respectively). This suggests that the transcriptional signals defining these states are less robust or more heterogeneous, which may reflect biological realities such as partial epithelial-mesenchymal transition, hybrid states, or low-frequency subpopulations that are harder to model with interpretable algorithms. Importantly, these findings not only validate the use of decision tree models for explainable cell classification but also underscore the inherent complexity of tumor cell dynamics.

Given that tumor progression often unfolds through specific, sequential transitions between closely related cell states, we refined our initial analysis by focusing on pairs of clusters that were directly connected along the pseudotime trajectory. Based on the inferred progression path 0 → 2 → 3 → 4, we aimed to identify genes and corresponding expression thresholds that may drive these lineage-specific transitions. To this end, we trained separate decision tree classifiers for each adjacent cluster pair: cluster 0 vs. 2, cluster 2 vs. 3, and cluster 3 vs. 4. This targeted, trajectory-guided approach improves both the interpretability and biological specificity of the results, enabling the identification of potential regulators of tumor evolution at higher resolution.

**FIGURE 7:**
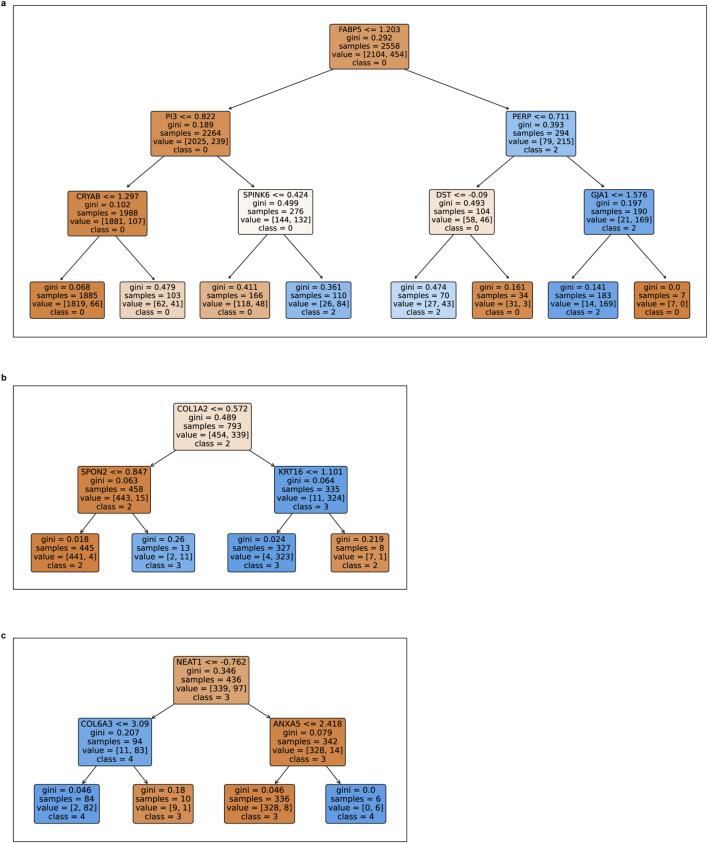
Decision trees highlighting key gene expression patterns across clusters. The trees illustrate the distinguishing features of cells in cluster pairs: 0 vs. 2 **(a)**, 2 vs. 3 **(b)**, and 3 vs. 4 **(c)**. Each node represents a gene and its corresponding expression threshold that optimally partitions the cells into distinct clusters, minimizing impurity and enhancing classification accuracy.

The resulting classification trees, illustrated in Figure 7, reveal distinct sets of gene-based decision rules that differentiate cell populations along each step of the trajectory. Each node in the tree represents a gene and its expression threshold that best separates the two compared cell populations, progressing from the root to the terminal leaves. The models were constrained to a maximum depth of 3, 2, and two for the respective cluster pairs, ensuring interpretability while retaining discriminatory power.

Below, we report the top decision rules derived from each model, highlighting the primary gene expression thresholds that distinguish the 2 cell populations. These top-level splits are particularly insightful, as they represent the most influential transcriptomic changes associated with each transition.

-Cluster transition: 0 → 2 (Figure 7a)

$$\text{Cluster}=\left\{\begin{array}{ll} 0, & \text{if FABP}5\leq 1.203\wedge \text{PI}3\leq 0.822\wedge \text{CRYAB}\leq 1.297\\ 2, & \text{if FABP}5>1.203\wedge \text{PERP}>0.711\wedge \text{GJA}1\leq 1.576 \end{array}\right.$$

Here, the root decision node is defined by FABP5, a gene associated with breast cancer cell survival, migration as well as other biological responses linked to fatty acid metabolism and inflammation (Pang et al., 2021). Additional discriminators include the inhibitor of elastase PI3 (elafin), and a member of the small heat-shock protein family as well as a molecular chaperone named CRYAB (Alpha B-Crystallin). Transition to cluster 2 is marked by increased FABP5 and PERP expression.

- Cluster transition: 2 → 3 (Figure 7b)

$$\text{Cluster}=\left\{\begin{array}{ll} 2, & \text{if COL}1A2\leq 0.572\wedge \text{SPON}2\leq 0.847\\ 3, & \text{if COL}1A2>0.572\wedge \text{KRT}16\leq 1.101 \end{array}\right.$$

The primary discriminator for this transition is COL1A2 (collagen type I alpha 1chain). Cluster 3 is further distinguished by elevated KRT16 (keratin 16), which is a well-acknowledged metastasis-associated protein that promotes EMT and motile features in breast cancer cells (Elazezy et al., 2021).

-Cluster transition: 3 → 4 (Figure 7c)

$$\text{Cluster}=\left\{\begin{array}{ll} 3, & \text{if NEAT}1>-0.762\wedge \text{ANXA}5\leq 2.418\\ 4, & \text{if NEAT}1\leq -0.762\wedge \text{COL}6A3\leq 3.090 \end{array}\right.$$

In this transition, NEAT1—a long non-coding RNA implicated in nuclear architecture and cancer progression—emerges as the most influential feature. The split highlights a reduction in NEAT1 and moderate expression of COL6A3 as key indicators of progression to cluster 4.

To enhance the generalizability and interpretability of these rules beyond a single dataset, in Supplementary Tables 2-7, we report them in relation to the empirical distribution of the observed values.

**FIGURE 8:**
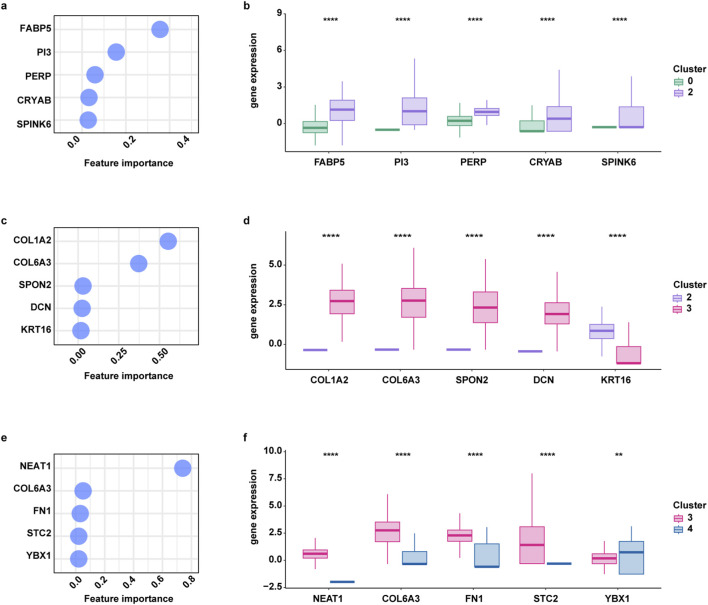
Main expression patterns influencing cellular population transitions. Feature importance of the five most relevant genes involved in cellular population transitions from cluster 0 to cluster 2 **(a)**, from cluster 2 to cluster 3 **(c)**, from cluster 3 to cluster 4 **(e)**. Box plots showing the differential expression of the features between cluster 0 and cluster 2 **(b)**, cluster 2 and cluster 3 **(d)**, cluster 3 and cluster 4 **(f)**. (****) indicates p < 0.0001. Gene expression comparisons were performed using the Wilcoxon rank-sum test.

Finally, to provide a deeper explanation of these decision patterns and to highlight crucial gene expression changes that drive cell state transitions, we focused our attention on the top five genes ranked by their feature importance (from high to low) for each couple of clusters (Figures 8a,c,e). Specifically, feature importance has been computed to quantify the relevance of each feature (or attribute) within tree-based models. This measure reflects how frequently and effectively a feature is used to split the data: features appearing near the root of the tree influence a larger fraction of samples and, consequently, have a stronger impact on the model’s predictions. For a given feature f, its importance (Breiman et al., 1984) is computed as:

$$\text{feature}‐\text{importance}\left(f\right)=\sum_{\begin{array}{c} t\;\epsilon \;T,\\ t\;split\;by\;f\; \end{array}}\frac{N_{t}}{N_{total}}\Delta I\left(t\right)$$

$$\text{where }\left\{\begin{array}{l} T:\text{set of all internal nodes in the tree}\\ N_{t}:\text{number of instances in }t\;\\ N_{total}:\text{total number of instances}.\;\\ \Delta I\left(t\right):\text{reduction of impurity }\\ \text{ achieved by the split at node }t \end{array}\right.$$

Finally, all feature importances are normalized so that they sum to 1.

Notably, we observed the upregulation of FABP5, PI3, PERP, CRYAB and SPINK6 in epithelial cells (cluster 2) respect to stem cells (cluster 0) (Figure 8b). Furthermore, mesenchymal stem cells (cluster 3) exhibited a significant downregulation of KRT16 but the upregulation of COL1A2, COL6A3, SPON2 and DCN respect to epithelial cells (cluster 2) (Figure 8d). Finally, elucidating the gene changes that characterize mesenchymal cell subtypes (cluster 3 to cluster 4), we found that NEAT1, COL6A3, FN1 and STC2 are downregulated in cluster 4 compared to cluster 3, while YBX1 is upregulated in cluster 4 respect to 3 (Figure 8f).

### 2.5 Multi-sample integration and identification of transition-driving genes

To strengthen the generalizability of our analytical pipeline, we applied the same workflow to an independent scRNA-seq dataset (GSE248288) obtained from four primary ER-positive breast tumors. Although our original sample represents a different clinical context, this analysis allowed us to test the broad applicability of our approach. We performed QC on each sample separately to account for sample-specific distributions of total counts, detected genes, and mitochondrial content, consistent with best practices in scRNA-seq analysis (Luecken and Theis, 2019; Stuart et al., 2019). All subsequent analytical steps were conducted as described in the Methods section, with the sole addition of batch correction using Harmony (Korsunsky et al., 2019) aimed at removing dataset-specific technical effects.

Cell-type annotation was performed by combining (i) canonical marker gene sets for different cell populations, (ii) automated scoring, and (iii) validation of cluster markers through the overlap between known canonical markers and cluster-specific DEGs, as reported in the Supplementary Table 1. This multi-layer annotation strategy ensured unbiased and reproducible cell-type identification across biological replicates. The analysis identified distinct epithelial, immune, and stromal populations (Supplementary Figure 1a).

Considering that we identified both epithelial and mesenchymal cells, we aimed to investigate whether an EMT may occur. Applying trajectory inference analysis as described before, we were able to reconstruct pseudotemporal progression and assess dynamic transcriptional changes along the EMT axis (Supplementary Figure 1b). As expected, the trajectory analysis revealed a continuous progression from epithelial to mesenchymal states, confirming the presence of EMT also in the explored dataset.

Next, a decision tree classifier with a maximum depth of 16 was trained on the entire dataset to model all classes, providing an overall perspective on the main transcriptional features that differentiate the diverse cell populations within the tumor. The resulting multi-class classification tree is shown in Supplementary Figure 2a. A zoom-in of its upper section reveals KRT18 as the root split that best separates the major cellular states, with subsequent nodes such as DCN and KRT8 providing finer discrimination among epithelial, fibroblast, and endothelial populations. The confusion matrix in Supplementary Figure 2b confirms strong classification performance across major cell types, while the ROC curves in Supplementary Figure 2c further demonstrate robust separability, consistent with the model’s overall accuracy of 87% and micro-average AUC of 93%.

We then focused on the classes involved in EMT to identify genes and expression thresholds that characterize this lineage-specific transition. Supplementary Figure 3 shows the resulting classification tree, whose depth was limited to four levels to balance interpretability with discriminatory power. By examining the first two splits from the root node and the genes that maximize information gain, we derived the following decision rules:

$$\text{Class}=\left\{\begin{array}{ll} Epithelial, & \text{if VIM}\leq 0.299\wedge \text{FN}1\leq 1.433\\ Mesenchymal, & \text{if VIM}>0.299\wedge \text{KRT}18\leq 0.555 \end{array}\right.$$

Consistent with the well-established role of VIM, FN1, and SNAI2 in driving the acquisition of a mesenchymal phenotype as well as the characteristic expression of keratin-encoding genes such as KRT8 and KRT18 in epithelial cells (Thiery and Sleeman, 2006), we found that our decision tree model relies on these genes. This indicates that the classifier can capture biologically meaningful signals: a high expression of mesenchymal markers is preferentially used to identify cells undergoing EMT-like transitions, whereas epithelial markers contribute to distinguishing cells that retain an epithelial identity. Interestingly, beyond these known markers, our model also identified additional genes with a potential discriminatory power. RASGRP2 emerged as a feature with markedly higher expression in mesenchymal cells, while ADTRP and AZGP1 were employed by the decision tree due to their lower expression in the mesenchymal state. Notably, the involvement of AZGP1 is particularly compelling: low AZGP1 levels have been previously associated with the promotion of EMT (Xu et al., 2016), aligning with the model’s use of its downregulation as a mesenchymal indicator. Taken together, these results indicate that the classifier not only relies on established lineage markers but also exploits novel potential features that further refine the separation between epithelial and mesenchymal identities.

Finally, to assess the robustness and generalizability of our approach, we performed a series of cross-dataset evaluation tests in which models trained on one dataset (our sample or the independent literature dataset) were used to classify cells in the other. The method consistently transferred across datasets, confirming its stability across independently generated data. Detailed results of these analyses are provided in the Supplementary Material.

## 3 Discussion

In our work, we investigated the dynamic transcriptional landscape of breast cancer at single-cell resolution by integrating pseudotime trajectory inference with interpretable machine learning. This approach enabled the identification of gene expression changes associated with transitions between distinct cellular states, providing insight into the regulatory mechanisms driving tumor progression.

Among the genes influencing cellular population transitions, we identified FABP5 (Fatty acid binding protein 5) as associated with the transition from stem-like to epithelial cells, and COL1A2 and COL6A3 as involved in EMT. These genes are of particular interest in the context of breast cancer, where dynamic transitions between cellular states are a hallmark of tumor progression (Park et al., 2010). In particular, FABP5 has been implicated in cancer cell proliferation, invasion, and poor prognosis in diverse breast cancer subtypes, and its expression may serve as a biomarker of aggressive, stem-like tumor cell populations (Pang et al., 2021). Similarly, COL1A2 and COL6A3, which are components of the ECM, have been implicated in the regulation of crucial cellular processes in breast tumors, including proliferation, metastasis, apoptosis, and drug resistance beyond EMT, thus shaping malignant progression and prognosis (Hendriks et al., 2024). Altogether, these findings indicate the genes involved in cellular population transition, such as FABP5, COL1A2, and COL6A3, as promising prognostic biomarkers and potential therapeutic targets in breast cancer, with implications for improving patient stratification toward personalized anti-cancer strategies.

Despite analyzing over 5,000 single cells from an infiltrating ductal carcinoma, a key limitation of our study lies in the absence of biological replicates. To address this limitation and increase the generalizability of our analytical approach, we applied our pipeline to an independent breast cancer scRNA-seq dataset from the literature. This complementary analysis confirmed the presence of epithelial and mesenchymal states—mirroring the patterns observed in our primary dataset—and demonstrated that our approach reliably captures EMT-related transcriptional dynamics, consistent with previous single-cell studies (Puram et al., 2017; Wu et al., 2021b; Wang et al., 2023). By validating our pipeline on an independent dataset, we demonstrate that the workflow can capture biologically meaningful transcriptional programs across samples, suggesting that the approach is robust and generalizable beyond the original dataset. Moreover, we conducted a series of cross-dataset evaluation tests to demonstrate the generalizability of our approach. Specifically, we showed that classification models trained on one dataset—whether derived from our sample or from the external literature dataset—perform consistently when applied to the other. This confirms that the proposed method is robust across independently generated datasets and capable of reliably classifying cell populations even when the training and testing data originate from different experimental sources. Future work will extend this framework to larger multi-sample cohorts to further validate and benchmark its robustness across diverse biological contexts.

Furthermore, while our primary goal was to identify expression patterns associated with cell state transitions, future work should include deeper functional analyses of the implicated genes to clarify their causal roles in tumor progression. Although our current analysis focused on a targeted panel of trajectory-associated marker genes, the approach can be readily extended to more comprehensive transcriptomic datasets, including whole-transcriptome or multi-omic profiles, in future studies.

In conclusion, our study demonstrates the power of combining high-resolution single-cell transcriptomics with interpretable machine learning to dissect the molecular dynamics of breast cancer. By applying decision tree classifiers to pseudotime-inferred transitions, we were able to uncover transparent, gene-level rules governing shifts between cellular states. This synergy between data-driven modeling and biological interpretability enables the identification of candidate regulators of tumor evolution and development of personalized therapeutic strategies in the context of precision oncology, even within the constraints of a single-patient dataset.

## 4 Methods

### 4.1 Tissue isolation and cell preparation

Breast cancer sample was collected from a 48-year-old woman with infiltrating ductal carcinoma who underwent surgical resection. The tumor was classified as an invasive carcinoma, moderately differentiated, composed of solid nests with occasional clear cells, associated with a moderate intraductal component, and without evidence of vascular invasion. Immunohistochemical analysis showed ER and PR-positivity, and a Ki67 proliferation index of 30%. The pathological stage was pT2N1. All procedures conformed to the Helsinki Declaration for the Research on Humans. Signed informed consent was obtained from the patient and the experimental research has been performed with the ethical approval provided by the “Comitato Etico Regione Calabria, Cosenza, Italy” – Approval code: 500/2022. The freshly excised specimen was placed in DMEM/F12 (Dulbecco’s modified Eagle’s medium) and transferred to the laboratory within 1 h of collection. The tissue sample was minced into approximately 1–2 mm^3^ pieces, washed with Dulbecco’s Phosphate-Buffered Saline (DPBS), and placed in Accumax cell detachment solution (Merck Life Science, Milan, Italy) for 45 min at room temperature with gentle but constant mixing. After letting the remaining tissue pieces settle down (for 2 min), the supernatant was filtered to obtain a single-cell suspension. Next, cells were centrifuged (300 rcf for 5 min). After discarding the supernatant, cells were resuspended into chilled 1× DPBS and centrifuged (300 rcf for 5 min). After discarding the supernatant, chilled 100% methanol was added drop by drop to 1 × 10^6^ cells that were stored at −80 °C for scRNA-seq library construction and sequencing.

### 4.2 scRNA-seq library construction and sequencing

The tube containing methanol-fixed cells, stored at −80 °C, was equilibrated to 4 °C for 5 min. Fixed cells were centrifugated at 1.000 rcf for 5 min at 4 °C. After the supernatant was removed without disrupting the cell pellet, an appropriate volume of Wash-Resuspension Buffer (0.04% BSA - 1 mM DTT - 0.2 U/ul RNase Inhibitor in 3X SSC Buffer) was added to obtain the desired cell concentration. Cell concentration was determined using a Countess II Automated Cell Counter (Thermo Fisher Scientific, Waltham, MA). The Trypan Blue staining of the methanol-fixed cells showed that 100% of the cells were dead, indicating that all cells were effectively fixed and permeabilized. For the library preparation and sequencing, Chromium Controller and Chromium NextGEM Single Cell 3′ Reagents Kit v3.1 (10X Genomics, Pleasanton, CA) have been used for partitioning cells into Gel Beads-in-emulsion (GEMs), where all generated cDNA shares a common 10x barcode. Libraries were generated from the cDNA following the manufacturer’s instructions. Enzymatic fragmentation and size selection are used to optimize the cDNA amplicon size. TruSeq Read 1 (read one primer sequence) is added to the molecules during GEM incubation. P5, P7, a sample index, and TruSeq Read 2 (read two primer sequence) are added via End Repair, A-tailing, Adaptor Ligation, and PCR. The final libraries containing the P5 and P7 primers were used in Illumina bridge amplification. After checking with both Qubit 2.0 Fluorometer (Invitrogen, Carlsbad, CA) and Agilent Bioanalyzer DNA assay (Agilent Technologies, Santa Clara, CA), libraries were prepared for sequencing and sequenced on paired-end 150 bp mode on NovaSeq6000 (Illumina, San Diego, CA). First, raw data was processed for both format conversion and de-multiplexing by Illumina BCL Convert v3.9.3 (Illumina, 2024). Thereafter, single Cell RNA-seq data processing was performed by processing of Chromium scRNA-seq output by Cell Ranger v 3.1.0 to align reads on reference genome (GRCh38) (Genomics, 2024). In particular, Cell Ranger count takes FASTQ files and performs alignment, filtering, barcode counting, and Unique Molecular Identifier (UMI) counting. It uses the Chromium cellular barcodes to generate feature-barcode matrices.

### 4.3 scRNA-seq data pre-processing

scRNA-seq data were processed using the Python-based toolkit Scanpy (v1.10.1) (Wolf et al., 2018), a scalable and modular framework optimized for large-scale single-cell datasets. The initial input consisted of a raw UMI (Unique Molecular Identifier) count matrix. To ensure the integrity of downstream analyses, we implemented a comprehensive data pre-processing pipeline including QC, normalization, feature selection, scaling, and dimensionality reduction.

The raw dataset contained 6,336 cells and 33,538 genes. Cells expressing fewer than 200 genes were removed to remove low-quality or empty droplets, while genes detected in fewer than 1% of all cells were excluded to reduce noise from non-informative features. In addition, cells exhibiting elevated mitochondrial gene expression—often indicative of stressed or apoptotic cells—were filtered out. We also removed cells with abnormally high total gene counts, a common signature of doublets or multiplets. To perform this, we applied a quantile-based filtering strategy, excluding cells below the second and above the 98th percentiles of total gene counts and mitochondrial gene proportions. After these QC steps, 5,834 high-quality cells and 11,747 genes were retained for downstream analysis.

Potential doublets, which can distort clustering and lead to spurious cell states, were identified using the Scrublet algorithm (Wolock et al., 2019), integrated into the Scanpy workflow. Scrublet simulates doublets and compares their gene expression profiles to the empirical dataset to flag probable doublet events. No cells in our dataset exceeded the threshold for doublet likelihood, indicating negligible doublet contamination.

Normalization was performed to correct for sequencing depth and technical variability. Using a global-scaling method, each cell’s total counts were normalized to 10,000 transcripts, followed by log-transformation. This approach facilitates comparability across cells while stabilizing the variance for downstream statistical analyses.

Genes exhibiting high cell-to-cell variability—often drivers of cell identity and state—were selected as highly variable genes (HVGs). These were identified by computing the variance-to-mean ratio for each gene and applying a loess-fit to distinguish technical from biological variation. The top 3,148 HVGs were selected, capturing the most informative features while minimizing the influence of stochastic noise from low-abundance genes.

To prepare the data for dimensionality reduction, gene expression values were standardized. Each gene was centered to have zero mean and scaled to unit variance. To mitigate the effect of extreme outliers, values were clipped at a maximum of 10 standard deviations. This scaling step ensures that highly expressed genes do not dominate the variance structure during principal component analysis.

### 4.4 Dimensionality reduction, clustering, and visualization

Dimensionality reduction was conducted using Principal Component Analysis (PCA), a linear method that captures the major axes of transcriptional variation. Fifty principal components (PCs) were retained based on the inflection point (“elbow”) in the scree plot, representing a balance between variance explained and dimensionality reduction.

When combining multiple biological samples, we applied batch correction using Harmony (Korsunsky et al., 2019) to mitigate dataset-specific technical variation while preserving underlying biological structure. Harmony was applied on principal component embeddings with batch identity as the covariate, and downstream analyses—including neighborhood graph construction, clustering, and visualization—were performed on the batch-corrected embeddings.

A k-nearest neighbor (k-NN) graph was constructed using the top 50 PCs, connecting each cell to its 10 nearest neighbors. This graph forms the backbone for both clustering and visualization. Uniform Manifold Approximation and Projection (UMAP) was applied for two-dimensional embedding, enabling visualization of the high-dimensional structure while preserving local and global relationships among cells.

For cell clustering, we employed the Leiden algorithm, a graph-based community detection method that optimizes modularity and is well-suited for scRNA-seq data (Traag et al., 2019). A resolution parameter of 0.3 was chosen based on silhouette analysis, which quantifies cluster cohesion and separation. This resolution represented the “elbow point” where further increases no longer substantially improved cluster quality. The resulting clusters were consistent with known cell-type structure and further validated by differential expression analysis.

### 4.5 Cell type annotation

The first step in cell annotation is to identify the DEGs, which enable the differentiation of various cell types or conditions by detecting genes that are significantly expressed differently between cell groups. To achieve this, we use the Wilcoxon rank-sum test, a non-parametric method that compares gene expression between two groups (Wilcoxon et al., 1970). By identifying DEGs, we can classify cells based on their gene expression profiles, revealing the molecular characteristics that define each cell type.

Cell type classification was carried out using the SCSA tool (Cao et al., 2020). It combines DEGs with confidence scores of cell markers derived from both established databases and user-defined inputs. The tool takes a DEGs cluster matrix and identifies marker genes for each cell cluster using log2 fold-change and p-value thresholds (LFC ⩾ 1, P < 0.05). Here, the p-value is used to assess the statistical significance of differential gene expression, ensuring that only genes with a high likelihood of true differential expression are considered as markers. SCSA integrates cell-type marker information from the CellMarker (Hu et al., 2022) and CancerSEA (Yuan et al., 2019) databases, both of which provide manually curated, experimentally validated markers for diverse human and mouse cell types. CellMarker encompasses over 20,000 markers across various tissues, while CancerSEA focuses specifically on cancer-related markers, annotating functional states across multiple cancer types.

### 4.6 Inference of cellular dynamics via pseudotime

To study dynamic changes in gene expression during cellular transitions, we performed downstream analyses following trajectory inference based on pseudotime. This approach allowed us to identify genes that are (i) associated with specific lineages along the inferred trajectory or (ii) differentially expressed between lineages, thereby uncovering the biological processes that drive cell state transitions.

Pseudotime trajectories were reconstructed using the Scanpy toolkit, applying the Diffusion Pseudotime (DPT) algorithm to model the differentiation patterns. This approach orders individual cells along a trajectory based on gradual changes in their gene expression profiles, even in the absence of explicit time-series data (Trapnell et al., 2014). To define the root of the trajectory, we selected the previously identified stem cell population, as these cells represent the earliest stage of the differentiation process. From this starting point, the trajectory unfolds, capturing branching events that correspond to potential lineage commitments.

For visualization purposes, we employed both UMAP to display the overall structure of the cellular landscape in a reduced-dimensional space, and Partition-based Graph Abstraction (PAGA) (Wolf et al., 2019) to represent the connectivity and probabilistic transitions between clusters. The combination of these tools enables an intuitive exploration of both local neighborhood relationships and the broader topology of cell-state transitions within the TME.

### 4.7 Decision tree modelling

To classify the 5,834 cells present in our sample, we trained a decision tree model (Breiman et al., 1984) on all clusters in the dataset, then refined the analysis by training models on specific cluster pairs based on trajectory inference results.

In all trials, the data was split into training and test sets with a 70/30 ratio to ensure sufficient training data while evaluating the model’s performance on unseen examples. Stratification on the target variable was applied to maintain the distribution of target labels across both sets. Unlike previous steps where dimensionality reduction techniques such as PCA were used, we performed classification using the raw count matrix, incorporating all available genes to avoid potential information loss associated with dimensionality reduction techniques. To label the dataset, we assigned the label obtained from the cell annotation step to each cell, ensuring that the full gene expression landscape was exploited. To obtain a reliable estimate of model performance, we employed k-fold cross-validation, partitioning the dataset into five stratified subsets and performing iterative training and testing across distinct data splits. This approach allowed us to assess the model’s generalization capability while mitigating overfitting. Classification performance was evaluated exclusively on the held-out test set at each fold using the standard metric accuracy (Han et al., 2012).

To further evaluate model performance, we computed confusion matrices and receiver operating characteristic (ROC) curves for each classification task (Fawcett, 2006). The confusion matrix provides a detailed breakdown of the model’s predictions by comparing the true labels of the test set with the predicted labels. Each element C [i, j] in the matrix represents the proportion of cells belonging to actual class i that were classified as class j. This metric enables the identification of specific misclassification patterns, offering insight into potential biological overlaps or ambiguities between closely related cell populations.

In parallel, we constructed ROC curves to assess the discriminative ability of the classifier across all classification thresholds. For multiclass tasks, ROC curves were computed in a one-vs-all manner, wherein each class was evaluated against all others combined. The area under the curve (AUC) was calculated for each class, serving as a summary measure of the model’s ability to distinguish between classes. Higher AUC values indicate better separability and robustness of the classifier, particularly in distinguishing biologically distinct cell states along the inferred tumor progression trajectory.

Model training was implemented using the Python Scikit-learn library (version 1.3.2) (Pedregosa et al., 2011). For cross-validation, the StratifiedKFold module was used, and classification trees were trained with the DecisionTreeClassifier module, choosing Gini as the criterion for the impurity measure (Breiman et al., 1984). The depth of the decision trees varied depending on the model being built and it was chosen by using the GridSearchCV function from the scikit-learn library in Python. The most important features were identified using the “feature_importances_” attribute of the DecisionTreeClassifier module in scikit-learn. Moreover, scikit-learn combines the proportion of samples influenced by each feature with the reduction in impurity achieved by splitting on that feature, producing a normalized estimate of its predictive contribution.

## Data Availability

The datasets presented in this study can be found in online repositories. The names of the repository/repositories and accession number(s) can be found below: https://www.ncbi.nlm.nih.gov/, GSE288223.
