# Supplementary material for: Integrating trajectory inference and self-explainable predictive models to explore cell state transitions in breast cancer at single-cell resolution
Source: Front Bioinform. 2026 Mar 4;6:1672671. doi: 10.3389/fbinf.2026.1672671 (PMC12996216; doi:10.3389/fbinf.2026.1672671)
Supplement: Supplementary file 1 [file Supplementaryfile1.docx]

***Supplementary Material***


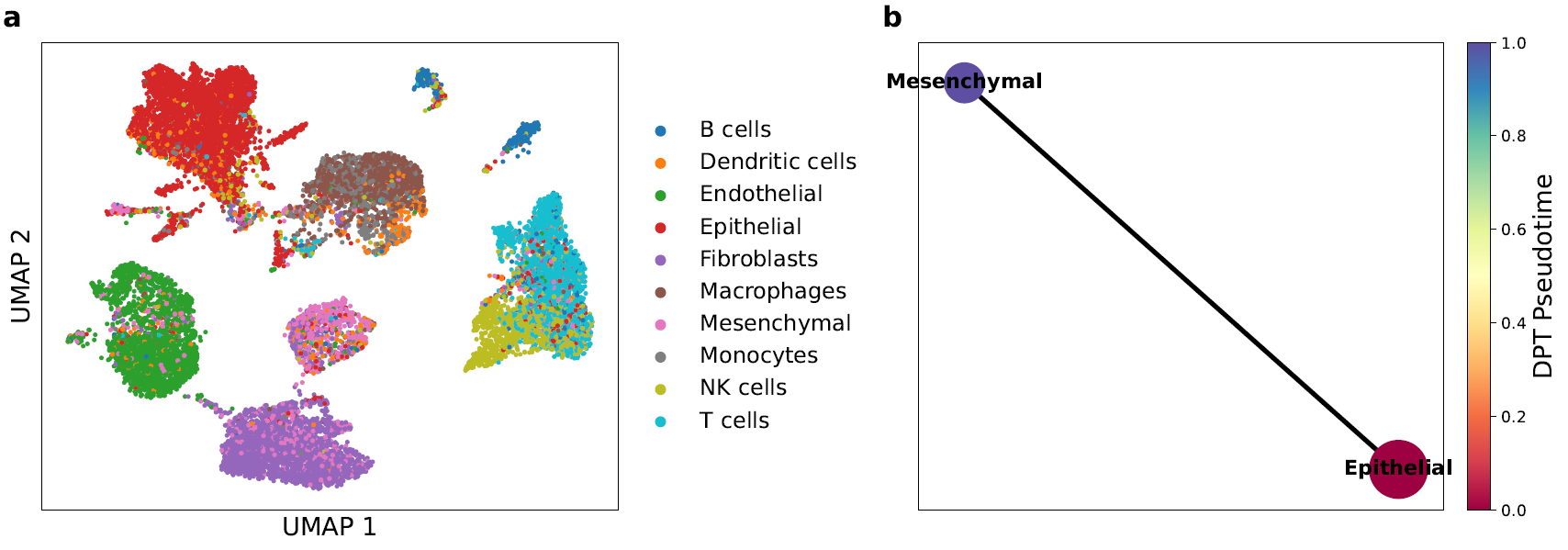


**Supplementary Figure 1.** Multi-sample integration and cell-state trajectory reconstruction. a) UMAP visualization of integrated single-cell data with cell-type annotations. The analysis identified distinct populations—including epithelial, mesenchymal, immune, endothelial, and fibroblast—based on canonical marker genes and cluster-specific differential expression. b) Trajectory inference analysis of epithelial and mesenchymal populations. Cells are ordered along normalized diffusion pseudotime (DPT) values, revealing a continuous progression from epithelial to mesenchymal states, consistent with an epithelial-to-mesenchymal transition (EMT).

| **Type** | **Markers Found in DEGs** |
| --- | --- |
| T cells | CD3D, CD3E, TRAC |
| B cells | MS4A1, CD79A |
| NK cells | GNLY, KLRD1, NKG7 |
| Monocytes | S100A9, S100A8, CD14, CD68, LYZ |
| Macrophages | CD163, CD68 |
| Dendritic cells | FCER1A, CST3 |
| Endothelial | CLDN5, VWF |
| Epithelial | KRT8, KRT18 |
| Fibroblasts | DCN, COL3A1, COL1A1 |
| Mesenchymal | FN1, VIM, SNAI2 |

**Supplementary Table 1.** Overlap between canonical marker genes and cluster-specific differentially expressed genes (DEGs). For each annotated cell type, the table lists the marker genes that were also identified as DEGs in the corresponding cluster, confirming the accuracy of cell-type annotation.


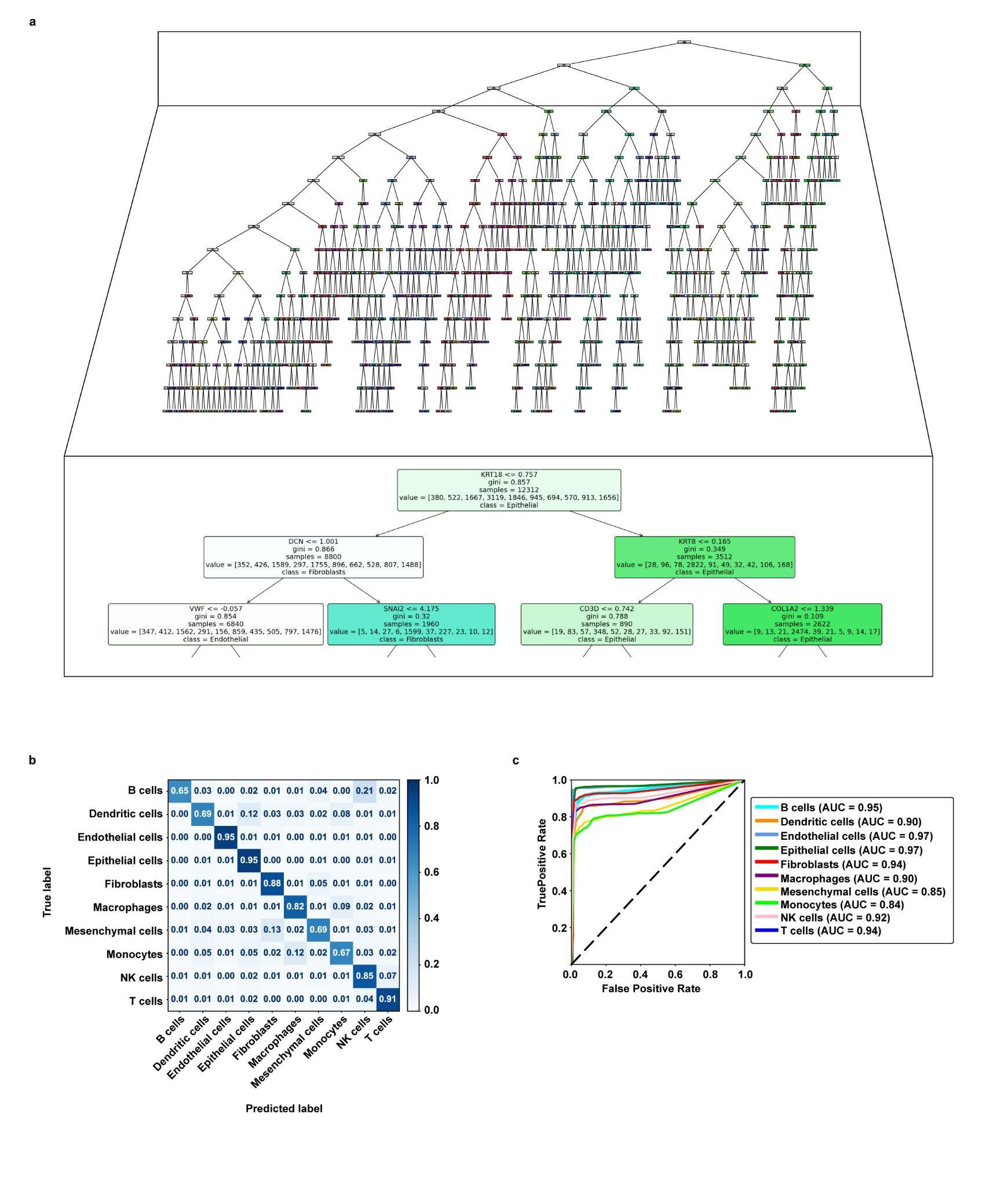


**Supplementary Figure 2.** Performance evaluation of the Decision Tree classifier on all cell types. a) The decision structure produced by the classifier, with a zoom-in of the upper portion of the tree, specifically the root node and the first two levels of the child nodes. b) Confusion matrix displaying the classifier’s performance by reporting, for each cell type, the percentage of correctly classified instances. c) ROC (Receiver Operating Characteristic) curves illustrating the model’s discriminative capability for each cell type.

**
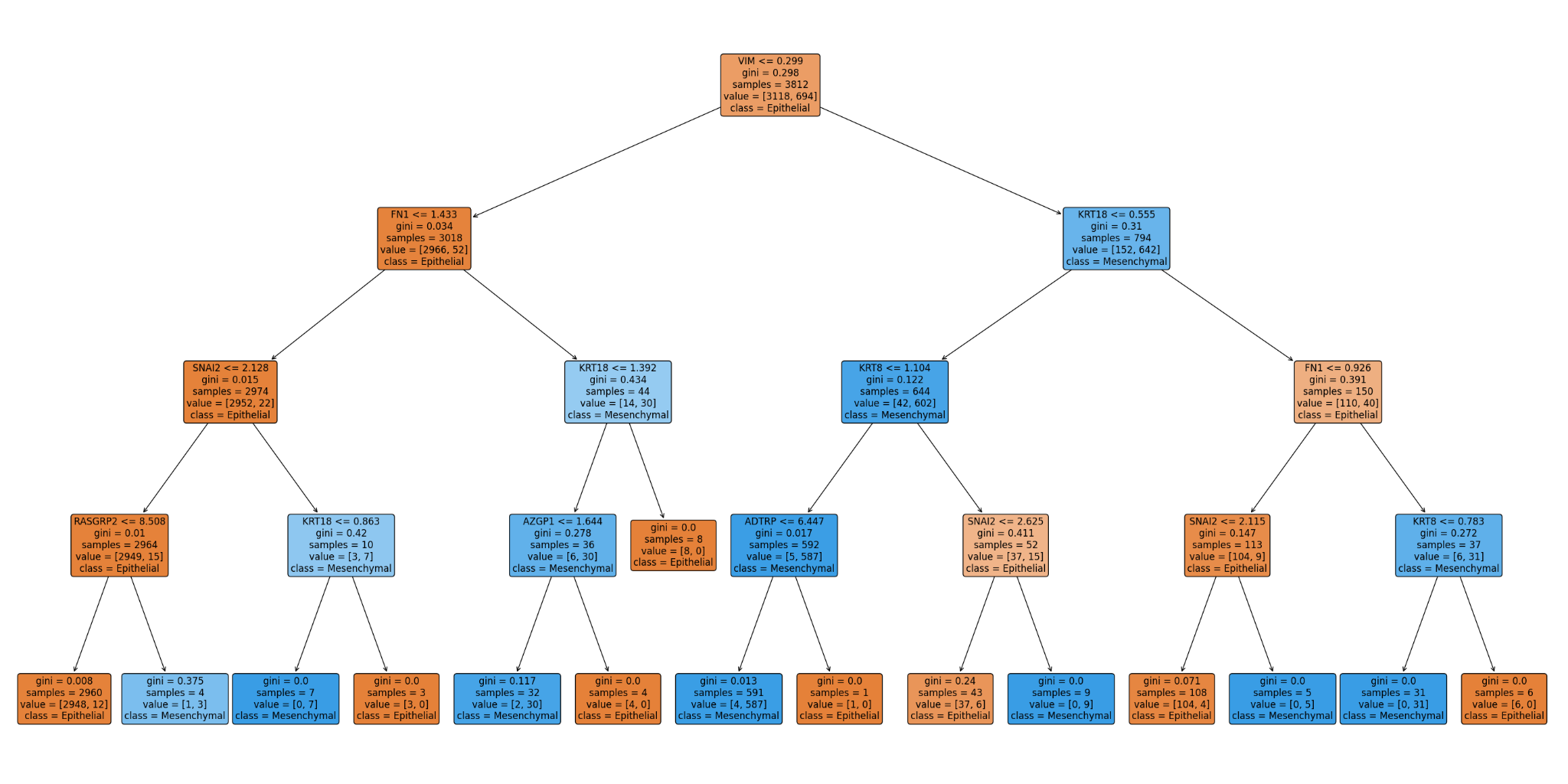
**

**Supplementary Figure 3.** Decision tree displaying key gene expression features associated with the transition from an epithelial to a mesenchymal state. By examining the first splits made on the highest nodes, it is evident that VIM is the gene that maximizes the information gain.

**Decision rules referred to cluster transitions**

The threshold values of the rules reported in Section 2.4, representing 0 → 2, 2 → 3 and 3 → 4 cluster transitions, are inherently dependent on the characteristics of the specific case study and on the statistical distribution of the corresponding genes. To enhance the generalizability and interpretability of these rules beyond a single dataset, we reformulated them in relation to the empirical distribution of the observed values. Specifically, we computed the five-number summary statistics, i.e. minimum, first quartile (Q1), median (Q2), third quartile (Q3), and maximum, for each gene involved in the rules. This reformulation enables the representation of rule thresholds in a distribution-aware manner, thereby mitigating dataset-specific bias and facilitating their application and comparison across different experimental contexts. The rules are reported in Supplementary Tables 2, 4 and 6, while the five-number summary statistics are reported in Supplementary Tables 3, 5 and 7, respectively.

| Cluster transition: 0 → 2 (Figure [7](#_bookmark7)a)  Rule n. 1  FABP5 <= 1.203 and PI3 <= 0.822 and CRYAB <= 1.297 🡪 class = 0  FABP5 < ${Max}^{FABP5}$ and PI3 < ${Max}^{PI3}$ and CRYAB < ${Max}^{CRYAB}$ 🡪 class = 0  Rule n. 2  FABP5 <= 1.203 and PI3 <= 0.822 and CRYAB > 1.297 🡪 class = 0  FABP5 < ${Max}^{FABP5}$ and PI3 < ${Max}^{PI3}$ and CRYAB > $Q_{3}^{CRYAB}$ 🡪 class = 0  Rule n. 3  FABP5 <= 1.203 and PI3 > 0.822 and SPINK6 <= 0.424 🡪 class = 0  FABP5 < ${Max}^{FABP5}$ and PI3 > $Q_{3}^{PI3}$ and SPINK6 < ${Max}^{SPINK6}$ 🡪 class = 0  Rule n. 4  FABP5 <= 1.203 and PI3 > 0.822 and SPINK6 > 0.424 🡪 class = 2  FABP5 < ${Max}^{FABP5}$ and PI3 > $Q_{3}^{PI3}$ and SPINK6 > $Q_{3}^{SPINK6}$ 🡪 class = 2  Rule n. 5  FABP5 > 1.203 and PERP <= 0.711 and DST <= -0.09 🡪 class = 2  FABP5 > $Q_{3}^{FABP5}$ and PERP <$Q_{3}^{PERP}$ and DST < $Q_{2}^{DST}$ 🡪 class = 2  Rule n. 6  FABP5 > 1.203 and PERP <= 0.711 and DST > -0.09 🡪 class = 0  FABP5 > $Q_{3}^{FABP5}$ and PERP < $Q_{3}^{PERP}$ and DST > $Q_{1}^{DST}$ 🡪 class = 0  Rule n. 7  FABP5 > 1.203 and PERP > 0.711 and GJA1 <= 1.576 🡪 class = 2  FABP5 > $Q_{3}^{FABP5}$and PERP > $Q_{2}^{PERP}$ and GJA1 < ${Max}^{GJA1}$ 🡪 class = 2  Rule n. 8  FABP5 > 1.203 and PERP > 0.711 and GJA1 > 1.576 🡪 class = 0  FABP5 > $Q_{3}^{FABP5}$ and PERP > $Q_{2}^{PERP}$ and GJA1 > $Q_{3}^{GJA1}$ 🡪 class = 0 |
| --- |

**Supplementary Table 2.** Decision rules representing 0 → 2 cluster transitions, and their reformulation with respect to gene quartile values involved in the rule.

| **Gene** | **Min** | **Q1** | **Q2** | **Q3** | **Max** |
| --- | --- | --- | --- | --- | --- |
| FABP5 | -1.7810514 | -0.6757632 | -0.22025654 | 0.47400591 | 3.462345 |
| PI3 | -0.5071806 | -0.5071806 | -0.5071806 | 0.29810629 | 6.8885593 |
| CRYAB | -0.6277369 | -0.6277369 | -0.6277369 | 0.35247636 | 5.7218614 |
| SPINK6 | -0.29783487 | -0.29783487 | -0.29783487 | -0.29783487 | 8.508516 |
| PERP | -1.7039555 | -0.07291945 | 0.34827328 | 0.75159453 | 2.6814034 |
| DST | -1.3028938 | -0.4047226 | 0.186282 | 0.67182675 | 3.177655 |
| GJA1 | -0.76090187 | -0.76090187 | 0.18823889 | 0.77469584 | 3.5628626 |

**Supplementary Table 3.** Minimum, first quartile (Q1), median (Q2), third quartile (Q3), and maximum, for each gene involved in the 0 → 2 rules.

| Cluster transition: 2 → 3 (Figure [7](#_bookmark7)b)  Rule n. 1  COL1A2 <= 0.572 and SPON2 <= 0.847 🡪 class = 2 (accuracy = 0.9910)  COL1A2 < $Q_{3}^{COL1A2}$and SPON2 < $Q_{3}^{SPON2}$ 🡪 class = 2 (accuracy = 0.9910)  Rule n. 2  COL1A2 <= 0.572 and SPON2 > 0.847 🡪 class = 3 (accuracy = 0.8461)  COL1A2 < $Q_{3}^{COL1A2}$ and SPON2 > $Q_{2}^{SPON2}$ 🡪 class = 3 (accuracy = 0.8461)  Rule n. 3  COL1A2 > 0.572 and KRT16 <= 1.101 🡪 class = 3 (accuracy = 0.9877)  COL1A2 > $Q_{2}^{COL1A2}$and KRT16 < ${Max}^{KRT16}$ 🡪 class = 3 (accuracy = 0.9877)  Rule n. 4  COL1A2 > 0.572 and KRT16 > 1.101 🡪 class = 2 (accuracy = 0.875)  COL1A2 > $Q_{2}^{COL1A2}$ and KRT16 > $Q_{3}^{KRT16}$ 🡪 class = 2 (accuracy = 0.875) |
| --- |

**Supplementary Table 4.** Decision rules representing 2 → 3 cluster transitions, and their reformulation with respect to gene quartile values involved in the rule.

| **Gene** | **Min** | **Q1** | **Q2** | **Q3** | **Max** |
| --- | --- | --- | --- | --- | --- |
| COL1A2 | -0.35668668 | -0.35668668 | -0.35668668 | 2.4920325 | 6.298936 |
| SPON2 | -0.3283491 | -0.3283491 | -0.3283491 | 2.0829992 | 6.7317877 |
| KRT16 | -1.1844056 | -0.6665069 | 0.3217688 | 1.007724 | 3.2885735 |

**Supplementary Table 5.** Minimum, first quartile (Q1), median (Q2), third quartile (Q3), and maximum, for each gene involved in the 2 → 3 rules.

| Cluster transition: 3 → 4 (Figure [7](#_bookmark7)c)  Rule n. 1:  NEAT1 <= -0.762 and COL6A3 <= 3.09 🡪 cluster = 4  NEAT1 <$Q_{1}^{NEAT1}$ and COL6A3 <$Q_{3}^{COL6A3}$ 🡪 cluster = 4  Rule n. 2:  NEAT1 <= -0.762 and COL6A3 > 3.09 🡪 cluster = 3  NEAT1 < $Q_{1}^{NEAT1}$ and COL6A3 > $Q_{2}^{COL6A3}$🡪 cluster = 3  Rule n. 3:  NEAT1 > -0.762 and ANXA5 <= 2.418 🡪 cluster = 3  NEAT1 > ${Min}^{NEAT1}$ and ANXA5 < ${Max}^{ANXA5}$ 🡪 cluster = 3  Rule n. 4:  NEAT1 > -0.762 and ANXA5 > 2.418 🡪 cluster = 4  NEAT1 > ${Min}^{NEAT1}$ and ANXA5 > $Q_{3}^{ANXA5}$ 🡪 cluster = 4 |
| --- |

**Supplementary Table 6.** Decision rules representing 3 → 4 cluster transitions, and their reformulation with respect to gene quartile values involved in the rule.

| **Gene** | **Min** | **Q1** | **Q2** | **Q3** | **Max** |
| --- | --- | --- | --- | --- | --- |
| **NEAT1** | -1.9757876 | -0.38241351 | 0.39655793 | 0.84943385 | 2.7074587 |
| **COL6A3** | -0.33204198 | 0.86132615 | 2.353306 | 3.36098085 | 6.082822 |
| **ANXA5** | -0.9588719 | 0.21853247 | 0.87066126 | 1.5851814 | 4.4674478 |

**Supplementary Table 7.** Minimum, first quartile (Q1), median (Q2), third quartile (Q3), and maximum, for each gene involved in the 3 → 4 rules.

**Cross-Dataset Classification Analysis**

To further assess the robustness and generalizability of our method, we performed cross-dataset classification analyses. We show that a model trained on dataset D1 (our sample) reliably classifies cells in dataset D2 (the independent literature dataset), and vice versa.

Before summarizing the approach, we note that D1 and D2 differ both in the number of detected genes (with partial overlap and dataset-specific genes) and in the set of annotated cell types (with some shared labels and some unique ones). Such differences are expected given that these two datasets originate from independent single-cell breast cancer studies, a disease characterized by strong heterogeneity, as well as by differences in experimental protocols, sample handling, sequencing depth, and data pre-processing. Despite these sources of variability, our cross-dataset tests demonstrate that classification models trained on one dataset can generalize well to the other.

Before running the tests, we performed a pre-processing step to ensure that both datasets shared the same feature structure. First, we identified the sets of genes and cell types common to the two datasets. We then filtered D1 and D2 to retain only these shared gene features and cell-type labels. Specifically, D1 originally contained 3,148 genes and D2 contained 2,000 genes; their intersection consisted of 386 genes, which we used to harmonize the feature space by reducing both datasets to this shared subset. An analogous procedure was applied to the cell-type annotations, resulting in two common labels, i.e., Epithelial and Mesenchymal, which were used as the target classes for the classification task.

After completing the pre-processing steps to ensure that both datasets had a homogeneous feature structure, we conducted a series of cross-dataset evaluation experiments. In these analyses, we trained the classification model on one dataset (used as the training set) and evaluated it on the other (used as the test set). The corresponding results are reported in Supplementary Table 8. Specifically, the Decision Tree classifier trained on dataset D1 and tested on dataset D2 achieved an accuracy of 91% and an AUC of 80%. Conversely, training the model on D2 and evaluating it on D1 yielded an accuracy of 91% and an AUC of 90%. These findings show that the classification models maintain strong predictive performance even when both the model and its learned gene–feature hierarchy are trained on one dataset and then applied to classify cells in the other. This demonstrates the generalizability and robustness of the proposed approach.

| **Training set** | **Test set** | **Tree Depth** | **Accuracy** | **AUC** |
| --- | --- | --- | --- | --- |
| D1 | D2 | 4 | 91% | 80% |
| D2 | D1 | 4 | 91% | 90% |

**Supplementary Table 8**. Performance evaluation of the Decision Tree classifiers, trained on the dataset D1(2) and tested on the dataset D2(1). The table reports the classifiers’ performance by reporting classification accuracy and AUC, showing the generalizability and robustness of the approach.
